# Molecular insights into sex-specific metabolic alterations in Alzheimer’s mouse brain using multi-omics approach

**DOI:** 10.1186/s13195-023-01162-4

**Published:** 2023-01-09

**Authors:** Abigail Strefeler, Maxime Jan, Manfredo Quadroni, Tony Teav, Nadia Rosenberg, Jean-Yves Chatton, Nicolas Guex, Hector Gallart-Ayala, Julijana Ivanisevic

**Affiliations:** 1 Metabolomics Unit, Faculty of Biology and Medicine, University de Lausanne, Lausanne, Switzerland; 2 Bioinformatics Competence Center, Faculty of Biology and Medicine, University de Lausanne, Lausanne, Switzerland; 3 Protein Analysis Facility, Faculty of Biology and Medicine, University de Lausanne, Lausanne, Switzerland; 4 Department of Fundamental Neurosciences, Faculty of Biology and Medicine, University of Lausanne, Lausanne, Switzerland

**Keywords:** Alzheimer’s disease, Multi-omics, Metabolomics, Lipidomics, Proteomics, 3xTg AD mouse, Sex differences, Amino acids, Lysophospholipids

## Abstract

**Background:**

Alzheimer’s disease (AD) is a progressive neurodegenerative disorder that is characterized by altered cellular metabolism in the brain. Several of these alterations have been found to be exacerbated in females, known to be disproportionately affected by AD. We aimed to unravel metabolic alterations in AD at the metabolic pathway level and evaluate whether they are sex-specific through integrative metabolomic, lipidomic, and proteomic analysis of mouse brain tissue.

**Methods:**

We analyzed male and female triple-transgenic mouse whole brain tissue by untargeted mass spectrometry-based methods to obtain a molecular signature consisting of polar metabolite, complex lipid, and protein data. These data were analyzed using multi-omics factor analysis. Pathway-level alterations were identified through joint pathway enrichment analysis or by separately evaluating lipid ontology and known proteins related to lipid metabolism.

**Results:**

Our analysis revealed significant AD-associated and in part sex-specific alterations across the molecular signature. Sex-dependent alterations were identified in GABA synthesis, arginine biosynthesis, and in alanine, aspartate, and glutamate metabolism. AD-associated alterations involving lipids were also found in the fatty acid elongation pathway and lysophospholipid metabolism, with a significant sex-specific effect for the latter.

**Conclusions:**

Through multi-omics analysis, we report AD-associated and sex-specific metabolic alterations in the AD brain involving lysophospholipid and amino acid metabolism. These findings contribute to the characterization of the AD phenotype at the molecular level while considering the effect of sex, an overlooked yet determinant metabolic variable.

**Supplementary Information:**

The online version contains supplementary material available at 10.1186/s13195-023-01162-4.

## Background

Alzheimer’s disease (AD) affects millions of lives as patients lose their cognitive functions and ability to live on their own [1, 2]. Despite many efforts, AD is still poorly understood, as evidenced by many failed clinical trials and the continued lack of treatments proven to stop neuronal death, although new treatments are currently being investigated [1, 3–5]. In addition, nearly twice as many women develop AD compared to men, but the reasons behind this sex bias remain unclear [1, 6]. External factors such as socioeconomic differences or the increased lifespan of women may play a role, but physiological differences have also been identified [1, 6–10]. Therefore, further insights into the molecular mechanisms underlying AD in men and women are clearly needed.

Omics-scale approaches provide a way to comprehensively explore molecular changes across multiple pathways, leading to a better understanding of the mechanisms that underlie complex diseases [11]. Applied to AD, omics studies have identified many alterations in metabolic pathways, particularly related to energy, lipid, and amino acid metabolism [12–34]. Interestingly, some of these changes were found to be exacerbated in females; notably, alterations in amino acid metabolism, the TCA cycle, and fatty acid metabolism were more pronounced in women [31–33]. In addition, mouse models of AD showed decreased mitochondrial respiration and earlier glucose hypometabolism in female brain tissue compared to males [33, 35, 36]. Sex differences in the regulation of metabolic processes might be at the origin of AD onset, and therefore need to be better characterized and explored.

The characterization and understanding of metabolic alterations underlying complex diseases such as AD can be improved through multi-omics approaches combining different layers of omics data [11, 37]. Since metabolites incorporate environmental input and represent the closest molecular link to the phenotype, metabolomics and lipidomics data complement other layers of omics data to enhance the functional understanding of a biological system in a physiologically healthy and diseased state [17–19, 38, 39]. The addition of other omics data layers is particularly important as genome-wide association studies alone have failed to explain the observed variability in AD [16, 40]. Multi-omics approaches have demonstrated that the combined effects of multiple molecular players (genes, proteins, metabolites) contribute to altered cellular and metabolic processes linked to AD pathology [17, 18, 39]. Therefore, the integration of multiple data layers to find colocalized and consistent changes in metabolic pathways can better explain the mechanisms underlying AD.

In an effort to generate multiple omics data layers from one sample and to integrate these data layers, classic approaches for sample preparation and data analysis need to be upgraded. A truly integrative approach would merge all layers of data for analysis, and therefore novel statistical approaches are often needed [37, 38, 41, 42]. Furthermore, inherent variability between omics data sets acquired on independent samples should be avoided, therefore methods that allow for the extraction and analysis of multiple small- and macro-molecule components of metabolism, from the same sample, should become a common practice [37]. An improved and integrative multi-omics workflow will allow for a better understanding of metabolic dysfunction at the level of the biochemical pathways that underlie the onset and progression of complex diseases.

We aimed to optimize a multi-omics approach relying on metabolomics (including lipidomics) and proteomics to identify potentially sex-biased metabolic pathways in the AD brain. To this end, we analyzed brain tissue lysate from the triple-transgenic AD (3xTg-AD) mouse (*APP* KM670/671NL, *PSEN1* M146V, and *MAPT* P301L mutations). This is a well-established model that exhibits most key pathologies, including the hallmark characteristics of amyloid-beta plaques and tau neurofibrillary tangles with aging, after the age of 12 months [43, 44]. Prior to these visible markers of cerebral AD pathology, the electrophysiological, morphological, and behavioral alterations were also identified at the age of 7–10 months in 3xTg-AD mice [45, 46]. Accordingly, for the present study, we chose to carry out experiments in mice aged 8 months which we hypothesized was likely to present alterations in the metabolic profile prior to appearance of established markers of AD pathology. To gain a comprehensive overview of metabolism, we first optimized a protocol to obtain high-coverage profiles of polar metabolites, lipids, and proteins from the same sample. We integrated the data obtained in an unsupervised model where we observed a clear sex bias within the disease. Integrative pathway analyses and lipid ontology analyses were performed to identify the metabolic pathways showing the strongest sex-biased alterations in AD. Our approach has led to the further characterization of sex differences in AD and can pave the way for future research into the molecular mechanisms underlying these sex biases.

## Methods

### Chemicals

Analytical grade acetonitrile, ammonium formate, formic acid, isopropanol, methanol, and water were purchased from Biosolve Chimie (FR), while ammonium acetate and acetic acid were purchased from Merck (USA).

### Samples

All experimental procedures were approved by the Veterinary Affairs Office of the Canton of Vaud, Switzerland, and were conducted as described previously by Van der Velpen et al. (2021) [47]. In short, male and female 3xTg-AD and WT mice were sacrificed at 8 months of age by decapitation without prior anesthesia in order to avoid alterations to the metabolic profile [47]. Whole brain was collected and immediately frozen on dry ice and stored at −80°C until the day of the analysis. Analyzed tissue samples consisted of five males and seven females per genotype. Whole brain tissue samples were mechanically homogenized under liquid nitrogen and aliquots were pre-weighed (~50 mg) in lysis tubes (Soft Tissue CK 14 Tubes, Bertin Technologies, USA).

### Sample preparation: polar metabolites, lipids, and proteins from brain tissue

#### Polar and lipid metabolite extraction

Pre-weighed homogenized brain tissue (~50 mg) was first extracted by the addition of 750 μl of methanol:water (4:1, v/v) solution with ceramic beads in the Cryolys Precellys 24 Sample Homogenizer (2 × 20s at 10,000 rpm and <10°C, Bertin Technologies, USA). Homogenized lysates were centrifuged for 15 min at 21,000 g and 4°C. 80 μl of the supernatant (polar metabolite extract) was collected for polar metabolite analysis. The remaining supernatant was collected and reserved. The residual pellet was re-extracted by the addition of 750 μl of 1-butanol:methanol (1:1, v/v), followed by homogenization and centrifugation as described above. The supernatant (lipid extract) was pooled with the reserved polar metabolite extract, evaporated to dryness under vacuum (Refrigerated CentriVap Concentrator coupled to CentriVap Cold Trap, Labconco, USA), and reconstituted in 350 μl of 1-butanol:methanol (1:1, v/v). The residual pellet was dried and stored on dry ice prior to protein extraction. Reconstituted extracts were vortexed for 30s, sonicated for 60s, and centrifuged for 15 min at 21,000 g and 4°C. Quality controls (QCs) were prepared by pooling 10 μl from each sample. A QC dilution series at 100, 50, 25, 12.5, and 6.25% was prepared for both polar and lipid metabolite analysis.

#### Protein preparation

Protein material after solvent extraction was resuspended in lysis buffer (1% Sodium deoxycholate, 30mM Tris pH 8.6, 10 mM DTT ) and re-homogenized in a FastPrep system. Aliquots of samples (100 μg at 2 μg/μl) were Cys-reduced/alkylated and digested following a modified version of the iST method [48], desalted on a Waters Oasis MCX plate, and dried. For peptide MS2 library construction, samples were mixed to create a pool, which was manually separated into 7 fractions by off-line basic reversed-phase (bRP) using the Pierce High pH Reversed-Phase Peptide Fractionation Kit (Thermo Fisher Scientific).

### LC-HRMS

#### Polar and lipid metabolite analysis

Polar and lipid metabolite extracts were analyzed by ultra-high-performance liquid chromatography (1290 UHPLC System, Agilent Technologies) coupled to a quadrupole time-of-flight mass spectrometer (6550 iFunnel Q-TOF LC/MS, Agilent Technologies) with an electrospray ionization source (Dual Agilent Jet Stream Electrospray Ionization) controlled by Agilent MassHunter Workstation Data Acquisition Software. ESI source conditions can be found in the supporting information.

Lipid analysis was carried out, in both positive and negative ionization modes, using a Zorbax Eclipse Plus C18 column (1.8 μm, 2.1 × 100 mm, Agilent Technologies) as described by Carrard and Gallart-Ayala et al. (2021) [49]. Briefly, mobile phases A and B consisted of 10 mM ammonium acetate and 0.1% acetic acid in acetonitrile:water (3:2, v/v) and isopropanol:acetonitrile:water (44:5:1, v/v) respectively. Flow rate was 0.6 ml/min, injection volume 2 μl, and column temperature 60°C. Separation of polar metabolites was carried out as described by Gallart-Ayala et al. (2018) [50]. Briefly, separation was performed for positive ionization mode using an Acquity UPLC BEH amide column (1.7 μm, 2.1 × 100 mm, Waters, IE). Mobile phase A consisted of 20 mM ammonium formate and 0.1% formic acid in water and mobile phase B consisted of 0.1% formic acid in acetonitrile. Flow rate was 0.4 ml/min, injection volume 2 μl, and column temperature 25°C. For negative ionization mode, separation was performed on a SeQuant ZIC-pHILIC column (5 μm, 2.1 × 100 mm, Merck, DE) preceded by a guard column (SeQuant ZIC-pHILIC Guard Kit, 2.1 × 20 mm, PEEK coated guard column, Merck, DE). Mobile phase A consisted of 20 mM ammonium hydroxide and 20 mM ammonium acetate in water and mobile phase B consisted of pure acetonitrile. Flow rate was 0.3 ml/min, injection volume 2 μl, and column temperature 30°C.

Full details on MS acquisition can be found in the supporting information. Briefly, sample data were acquired in full scan acquisition mode in the range of 50–1700 *m/z* at a rate of 2 spectra/s. QCs at the beginning of the analytical run were acquired by tandem mass spectrometry through iterative data-dependent acquisition over five iterations with rolling exclusion, in the range of 30–1700 *m/z* at a rate of 3 spectra/s with a narrow isolation width (~1.3 amu). Different collision energy conditions were tested on tissue extracts to determine the optimal conditions for the highest coverage (Figure S1). As a result, we concluded that the combined 25–40 eV for lipidomics analysis in both positive and negative mode, 10–25 eV for metabolomics analysis in positive ionization mode, and 10 eV for metabolomics analysis in negative ionization mode provided the best coverage.

QCs and QC dilution series were analyzed at the beginning and end of analytical runs. QCs were further injected regularly between every ten samples throughout the overall analytical run.

#### Protein analysis

To profile brain proteomes we applied a Data-Independent Acquisition (DIA) strategy. LC-MS/MS analysis was carried out on a TIMS-TOF Pro (Bruker, Bremen, Germany) mass spectrometer interfaced through a nanospray ion source to an Ultimate 3000 RSLCnano HPLC system (Dionex). Peptides were separated on a reversed-phase (custom packed) 40 cm C_18_ column (75 μm ID, 100Å, Reprosil Pur 1.9 μm particles, Dr. Maisch, Germany) at a flow rate of 0.250 μl/min with a linear 6–45% acetonitrile gradient in 107 min. Identical LC gradients were used for data-dependent and data-independent acquisition (DDA resp. DIA using PASEF) measurements using standard MS methods as published [51]. Detailed description of the instrument and data processing parameters can be found in the supporting information.

### Data processing

Metabolomics and lipidomics data were converted to ABF file format and uploaded to MS-DIAL software (ver. 4.48) for peak detection, chromatogram alignment, and peak annotation [52, 53]. Polar and lipid metabolites were annotated based on accurate mass and MS/MS spectral matching using, respectively, the Fiehn HILIC and LipidBlast mass spectral libraries [54, 55]. Polar metabolites were further annotated based on accurate mass and MS/MS spectral matching using METLIN Cloud spectral library (https://metlincloud2.massconsortium.com/) [56, 57]. The peak area for each annotated metabolite was taken for statistical analyses.

Metabolomics and lipidomics data were normalized to sample tissue weight (Table S6 and S7). The systematic bias in peak area over time (signal intensity drift) inherently present in LC-MS data was corrected using an adaptive cubic smoothing spline algorithm (LOWESS) on pooled QC samples injected regularly between samples over the course of the run [58]. Positive linearity across the QC dilution series was visually inspected and metabolites (including lipids) with a coefficient of variation > 30% across QCs and/or *r*^2^ < 0.65 were discarded. Metabolomics and lipidomics data were log_10_ transformed prior to analysis.

For protein identification and quantitation by DIA, Bruker MS data were processed with Spectronaut 14.10 (Biognosys, Schlieren, Switzerland). A library was constructed from the DDA data for the bRP fractions by searching the reference mouse proteome (RefProt, www.UNIPROT.org) (55485 sequences). The library contained 7951 protein groups. Peptide-centric analysis of DIA data with Spectronaut used the library described above and standard parameters, leading to the identification and quantitation of 6742 protein groups. Exported protein group intensities normalized by Spectronaut were further analyzed with Perseus [59]. Only protein groups quantitated by on average 3 precursors were kept (5115 proteins — see Table S8). Intensities were log_2_-transformed and Welch *t*-tests were computed to compare all conditions.

### Statistical analysis

The significant effects of genotype, sex, and their interaction (sex:genotype) on polar metabolites, lipids, and proteins were tested using an ANOVA with a non-sequential sum of square (type III) to deal with the unbalanced design between males and females. Polar metabolites and lipids were log10 transformed to normalize their error distribution. *P*-values for the effects of sex, genotype, and sex:genotype were adjusted for multiple testing using the Benjamini & Hochberg procedure to control for false discovery rate (FDR).

Multi-omics analysis was performed using Multi-Omics Factor Analysis (MOFA2 version 1.2.0) [60]. Polar metabolites, lipids, and proteins datasets were further transformed independently using EigenMS [61] to improve dataset normalization and reduce the undesired source of variation as suggested by MOFA authors. MOFA model training was started with 8 factors, 0.005 variance explained threshold for factor drop and “slow” convergence mode of the ELBO. Other options were set as default.

#### Pathway and enrichment analyses

Integrative analysis of proteins and polar metabolites was performed using the online MetaboAnalyst software (ver. 4.0 and 5.0) [62]. Proteins and polar metabolites with a significant raw *p*-value for interaction effect were uploaded for Joint Pathway Analysis. The following analysis parameters were chosen: hypergeometric test for enrichment analysis, degree centrality for topology measure, and combining *p*-values weighted at the pathway level for integration.

Lipid ontology analysis was performed using the online LION/web Lipid Ontology Enrichment software [63]. Lipid species and their corrected *p*-value for genotype effect were uploaded for ranked analysis.

Protein enrichment in KEGG was first performed by mapping uniprot ID to keggid and keggid to KEGG pathways using the KEGGREST R package (v1.32.0). KEGG pathways enrichment was then performed using a rank sum test or a hypergeometric test. Background universe for enrichment analysis contained all proteins that were quantified. Individual proteins involved in lysophospholipid metabolism were found by matching identifiers with UniProt results for the keywords “phospholipase A1,” “phospholipase A2,” “lysophospholipid acyltransferase,” and “lysophospholipase” [64]. Matches were manually curated based on function.

#### Data visualization

Graphs were made in Python. Boxplots were generated from fold-change values where the abundance of a given compound in a sample was normalized to the median male WT value for the said compound. This was done to have consistent axes but does not change the distribution. Given the broad range of compound abundances, data was standardized prior representation: first, standard scores were calculated for each compound in a sample using the sample mean and standard deviation (x-μ)/σ. Then the median score for a given group (male or female, WT or 3xTg-AD) was used to generate heatmaps.

## Results

### From simultaneous extraction of polar metabolites, lipids, and proteins to multi-omics analysis

**Fig. 1:**
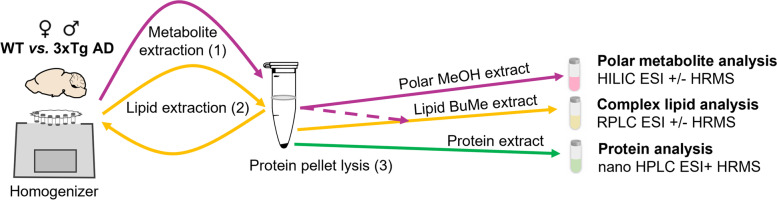
Extraction pipeline to obtain multiple data layers from the same sample. A sequential extraction method followed by an untargeted mass spectrometry approach was used to obtain multiple data layers from the same samples of whole brain homogenate (50 mg) from male and female WT and 3xTg-AD mice. A first extraction (pink arrows) was performed with methanol:water (4:1, v/v), and an aliquot of the resulting supernatant was analyzed for polar metabolites. The same sample was extracted a second time with BuMe (butanol:methanol 1:1, v/v, yellow arrows), and the pool from both organic solvent extractions was evaporated, reconstituted and analyzed for complex lipids. Proteins from the remaining pellet were extracted and digested by a modified iST method (1% Na+DCA, 30mM Tris 10 mM DTT), then analyzed (green arrows). Omics data were acquired using LC-ESI-HRMS. Using MS/MS spectral matching, 119 polar metabolites, 600 unique lipid species, and 5115 proteins were annotated with high confidence. WT: wild-type; 3xTg-AD: triple transgenic Alzheimer’s disease model; LC: liquid chromatography; ESI: electrospray ionization; HRMS: high resolution mass spectrometry; MS/MS: tandem mass spectrometry

With the goal of integrating metabolomics, lipidomics, and proteomics data, we aimed to reduce variability between these datasets as much as possible. Therefore, as opposed to using independent samples to extract each data layer, we developed a sequential extraction protocol to extract polar metabolites, complex lipids, and proteins from the same sample while relying on single-phase extraction methods. The first step consisted of the extraction of polar metabolites using a methanol:water (4:1 v/v) solution (Fig. 1). An aliquot of this extract was analyzed by hydrophilic interaction chromatography coupled to high-resolution mass spectrometry (HILIC-HRMS) for the measurement of polar and moderately polar metabolites. Complex lipids were recovered from the remaining pellet by a subsequent extraction with 1-butanol:methanol (1:1 v/v, BuMe) (Fig. 1) [65, 66]. For maximized coverage of amphipathic and non-polar lipids, methanolic and BuMe extracts were pooled, pre-concentrated, and analyzed using reversed phase liquid chromatography (RPLC) coupled to HRMS. Finally, the precipitated protein pellet was used for proteomics analysis with nanoLC-HRMS (Fig. 1). The content of each acquired data layer (e.g., polar metabolome, lipidome, and proteome), was characterized by matching against either experimentally acquired or in silico created spectral libraries of tandem mass spectrometry (MS/MS) data (see Materials and Methods for detailed description). As a result, we identified a comprehensive panel of metabolites and proteins, consisting of 119 polar metabolites, 600 unique lipid species, and 5115 reliably quantified proteins, which constitute a molecular signature of mouse brain tissue (Table S1–S3).

**Fig. 2:**
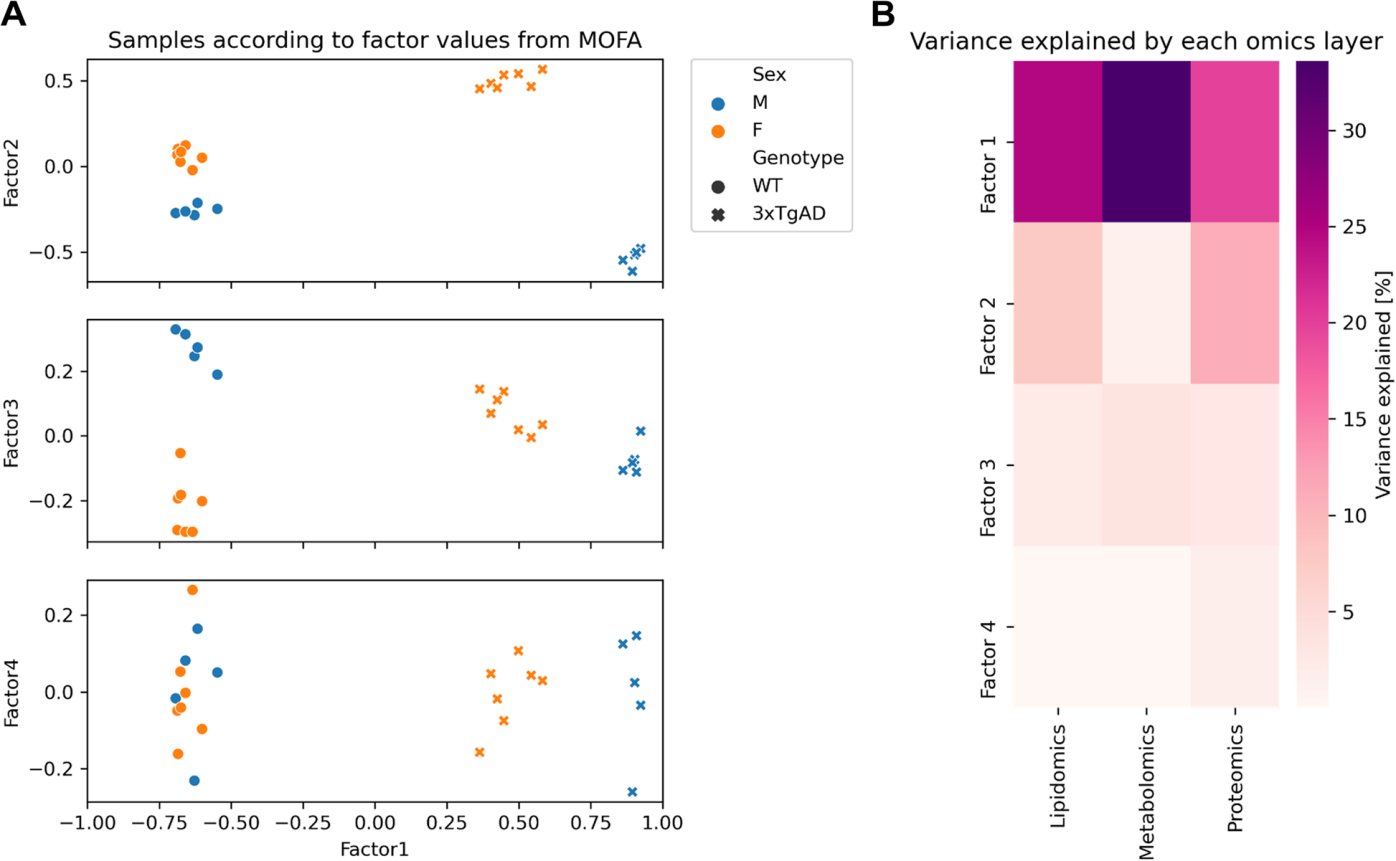
Multi-omics factor analysis. **A** Samples plotted according to factor values from MOFA. Male and female animals are represented by the colors blue and orange, respectively. WT and 3xTg-AD animals are represented by circles and crosses, respectively. **B** Percent of variance explained by each omics data layer per MOFA factor. MOFA, multi-omics factor analysis; M, male; F, female; WT, wild-type; 3xTg-AD, triple transgenic Alzheimer’s disease model

This multi-layered molecular signature was explored using Multi-Omics Factor Analysis (MOFA) to identify the dimensions (i.e., factors) that capture the joint or specific source of variation within the analyzed omics set of 3xTg-AD and wild-type (WT) animals [60]. In parallel, we calculated the significant effect of genotype, sexes, and sex:genotype interaction for each molecular species (Table S1–S3). As illustrated in Fig. 2A, the metabolic profiles of the polar metabolome, lipidome, and proteome showed a strong sample clustering by genotype and sex. In particular, Factor 1 captured the difference between 3xTg-AD and WT genotype, with a small sex bias within the 3xTg-AD group (Fig. 2A). This factor explained the largest part of the variance among all datasets (Fig. 2B). Accordingly, we observed that Factor 1 top contributors have a significant effect of genotypes (Figure S2 Factor 1). Factor 2 identified molecular features with weak differences between male and female under WT condition but that strongly diverge under 3xTg-AD. Top contributors to Factor 2 displayed a large genotype effect carried by the WT female response (set as the reference) with a strong sex:genotype interaction that reduces or inverts this response in males, nicely highlighted by lipidomics data (Figure S2 Factor 2). Although variance in proteomics data could be explained by Factor 2, this response was surprisingly absent in the annotated mouse metabolome (Fig. 2) which suggests that this response is either lipid-specific or beyond the coverage of our methods. Factor 3 captured a smaller part of variance that is opposed to Factor 2. A strong sex difference is observed in WT mice, but this difference disappeared under 3xTg-AD. However, because of the smaller effect sizes within this factor, most of these data do not pass the significant FDR threshold of our linear model (Figure S2 Factor 3). Finally, Factor 4 revealed some unknown small source(s) of variation within our dataset.

### Integrative enrichment analysis reveals sex-biased amino acid metabolism in the triple-transgenic AD mouse brain

**Fig. 3:**
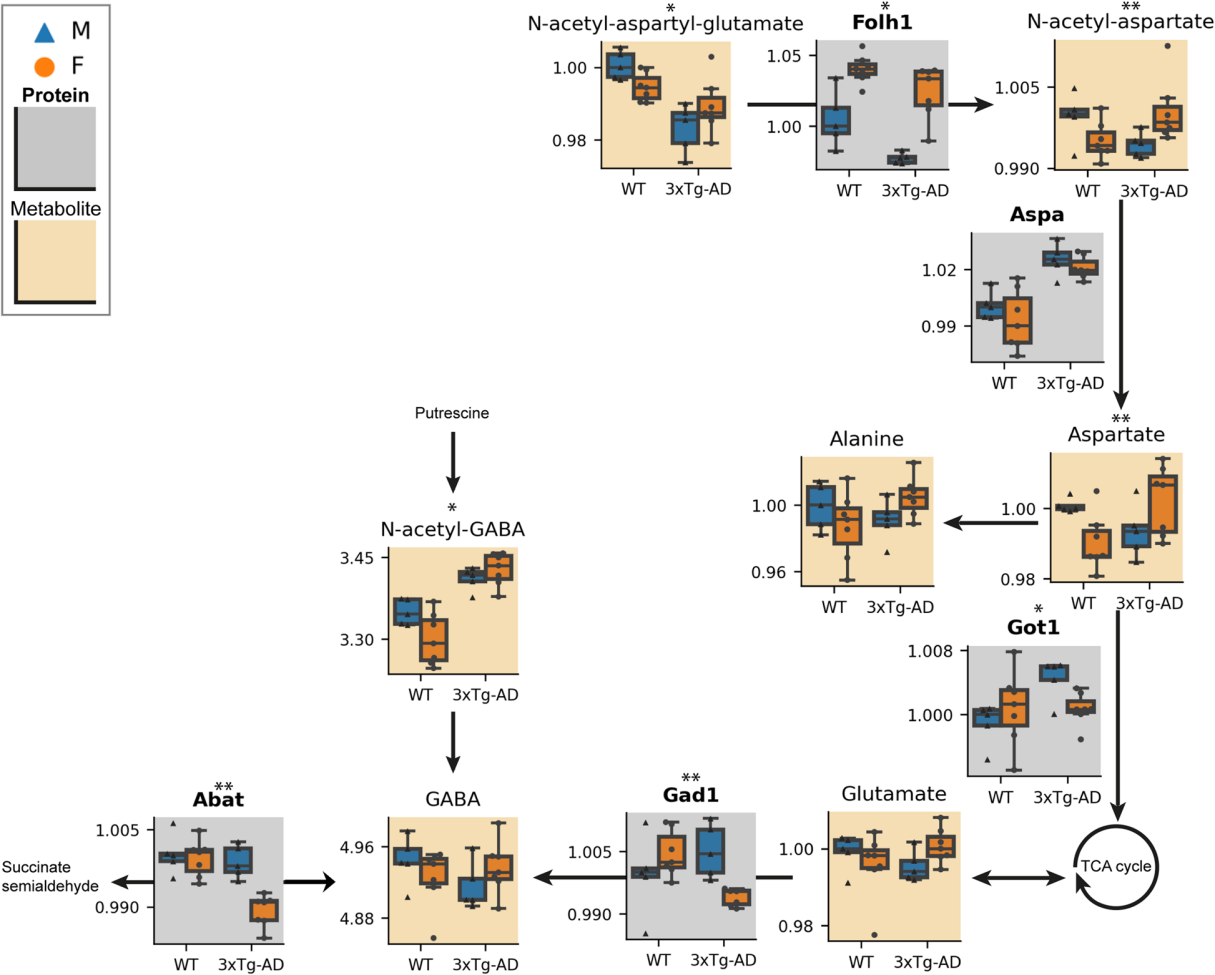
Interaction effects in the Alanine, Aspartate, and Glutamate metabolic pathway. Selected measured metabolites (beige background) and proteins (gray background, bold title) from the alanine, aspartate, and glutamate metabolic pathway are represented by boxplots. Each line of the boxplot is a quartile of the compound abundancy per sample group (relative to the median value of male WT). Boxes are colored blue or orange with triangular or circular points for males and females, respectively. Asterisks above plots denote significant *p*-values for a sex:genotype interaction effect, less than 0.05 (*) or less than 0.01 (**). M, male; F, female; WT, wild-type; 3xTg-AD, triple transgenic Alzheimer’s disease model

Following MOFA analysis, the revealed significant alterations from the top Factors were mapped onto biochemically relevant pathways using integrative enrichment analysis. Within amino acid metabolism, both the alanine, aspartate, and glutamate pathway and the arginine biosynthesis pathway were found to be significantly affected in the AD mouse model in a sex-dependent fashion (Table S4). The alanine, aspartate, and glutamate pathway exhibited two areas of coordinated sex-biased changes upon AD (Fig. 3). Metabolites implicated in the conversion of N-acetyl-aspartyl-glutamate (NAAG) to aspartate (i.e., N-acetyl-aspartyl-glutamate, N-acetyl-aspartate, and aspartate) showed consistently lower levels in male 3xTg-AD compared to WT, while this decrease was not seen in females (Fig. 3) (interaction effect, p_N-acetyl-aspartyl-glutamate_ = 0.019, p_N-acetyl-aspartate_ = 0.008, p_Aspartate_ = 0.007). Accordingly, the expression of the Folh1 enzyme which initiates this conversion was also downregulated in males (interaction effect p_Folh1_ = 0.021). Changes were also observed in the part of the pathway associated with the synthesis of gamma-aminobutyric acid (GABA). The enzymes Abat and Gad1, responsible for its synthesis, were downregulated in 3xTg-AD females but not in males (Fig. 3) (interaction effect, p_Abat_ = 0.006, p_Gad1_ = 0.004). However, total GABA levels were not significantly changed in any of the populations (Table S2). On the other hand, the intermediate metabolite N-acetyl-GABA, derived from putrescine through a different pathway, was significantly increased in the 3xTg-AD animals compared to WT (genotype effect p_N-acetyl-GABA_ = 0.005) (Fig. 3). Finally, although other individual enzymes of the alanine, aspartate, and glutamate metabolic pathway also showed genotype- and sex-biased expression, the metabolite data is not sufficient to explain their downstream effects (Fig. 3, Figure S3).

**Fig. 4:**
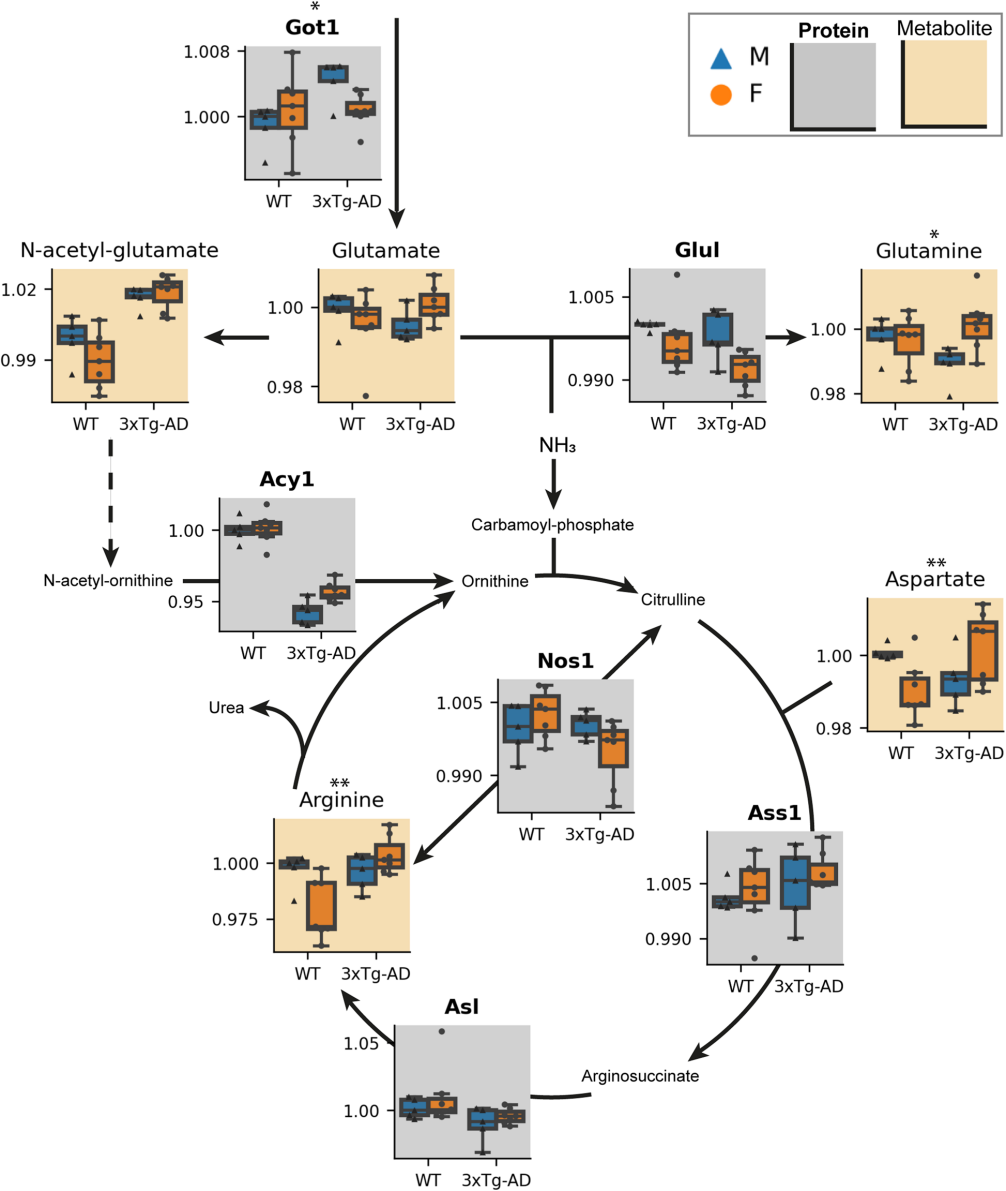
Interaction effects in the Arginine biosynthesis pathway. Measured metabolites (beige background) and proteins (gray background, bold title) from the arginine biosynthesis pathway are represented by boxplots. Each line of the boxplot is a quartile of the compound abundancy per sample group (relative to the median value of male WT). Boxes are colored blue or orange with triangular or circular points for males and females, respectively. Asterisks above plots denote significant *p*-values for a sex:genotype interaction effect, less than 0.05 (*) or less than 0.01 (**). M, male; F, female; WT, wild-type; 3xTg-AD, triple transgenic Alzheimer’s disease model

The second significantly enriched pathway was arginine biosynthesis (Fig. 4). Here, however, we observed fewer coordinated hubs of sex-biased alterations. Nonetheless, it is interesting to note an increase in arginine levels in 3xTg-AD females compared to WT, but not in males (interaction effect p_Arginine_ = 0.006). Similarly, the expression of neighboring enzyme Nos1 was lower in 3xTg-AD females compared to WT (p_Nos1_ = 0.031). We also observed genotype differences in this pathway (Table S1–S3), notably, decreased levels of Glul (genotype effect p_Glul_ = 0.036), essential for the elimination of ammonia as glutamine in the healthy brain, in 3xTg-AD mice.

### Lipid ontology enrichment analysis reveals altered fatty acid and lysophospholipid metabolism in the triple-transgenic AD mouse brain

**Fig. 5:**
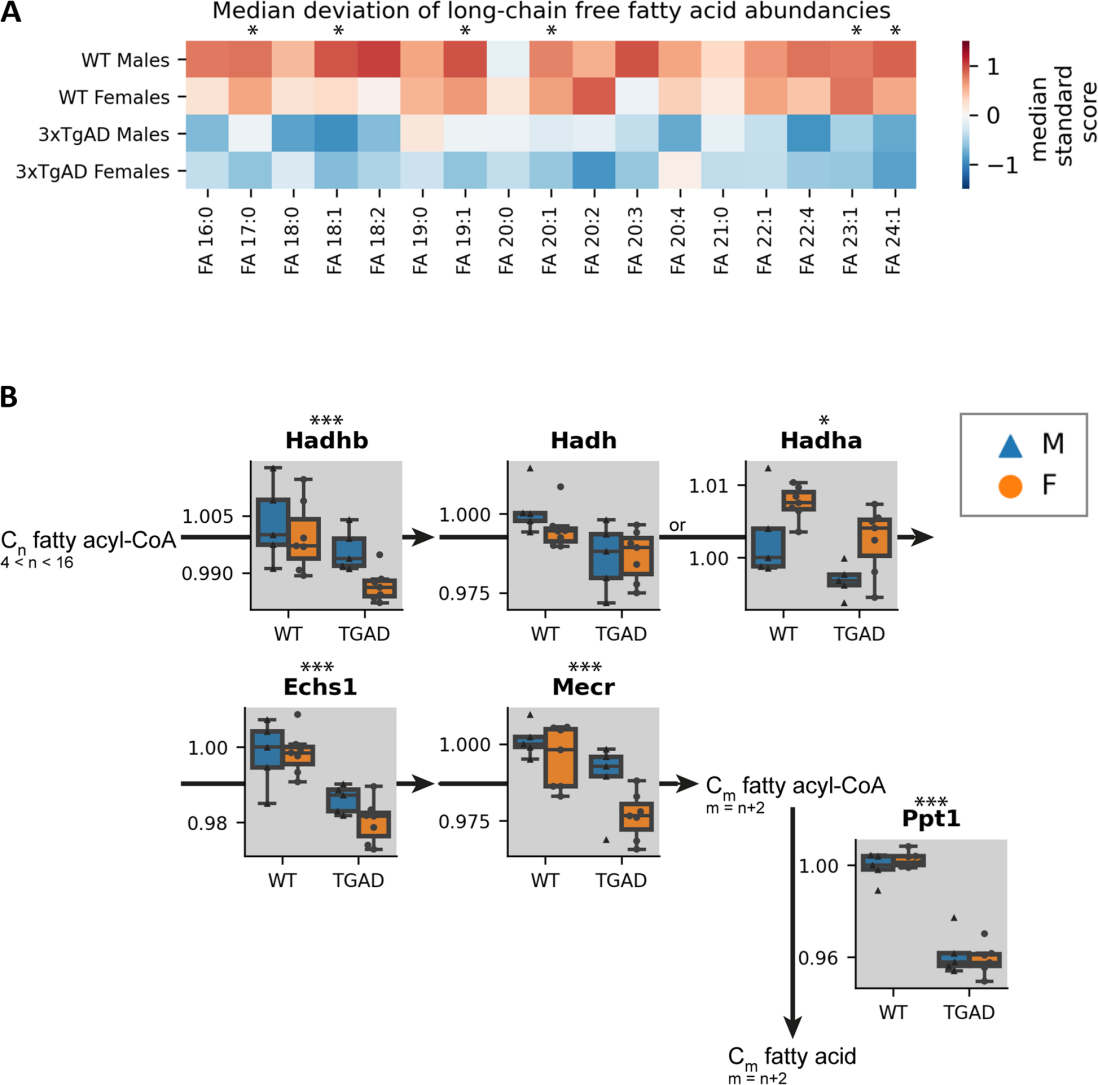
Genotype effects in long-chain free fatty acids. **A** Heatmap of median standard scores of long-chain free fatty acid species abundance in each sample group. **B** Schema of free fatty acid elongation in the mitochondria. Proteins are represented by boxplots where each line of the boxplot is a quartile of the compound abundancy per sample group (relative to the median value of male WT). Boxes are colored blue or orange with triangular or circular points for males and females, respectively. Asterisks above plots denote significant *p*-values for a genotype effect, less than 0.05 (*), less than 0.01 (**), or less than 0.001 (***). FA, free fatty acid; M, male; F, female; WT, wild-type; 3xTg-AD, triple transgenic Alzheimer’s disease model

**Fig. 6:**
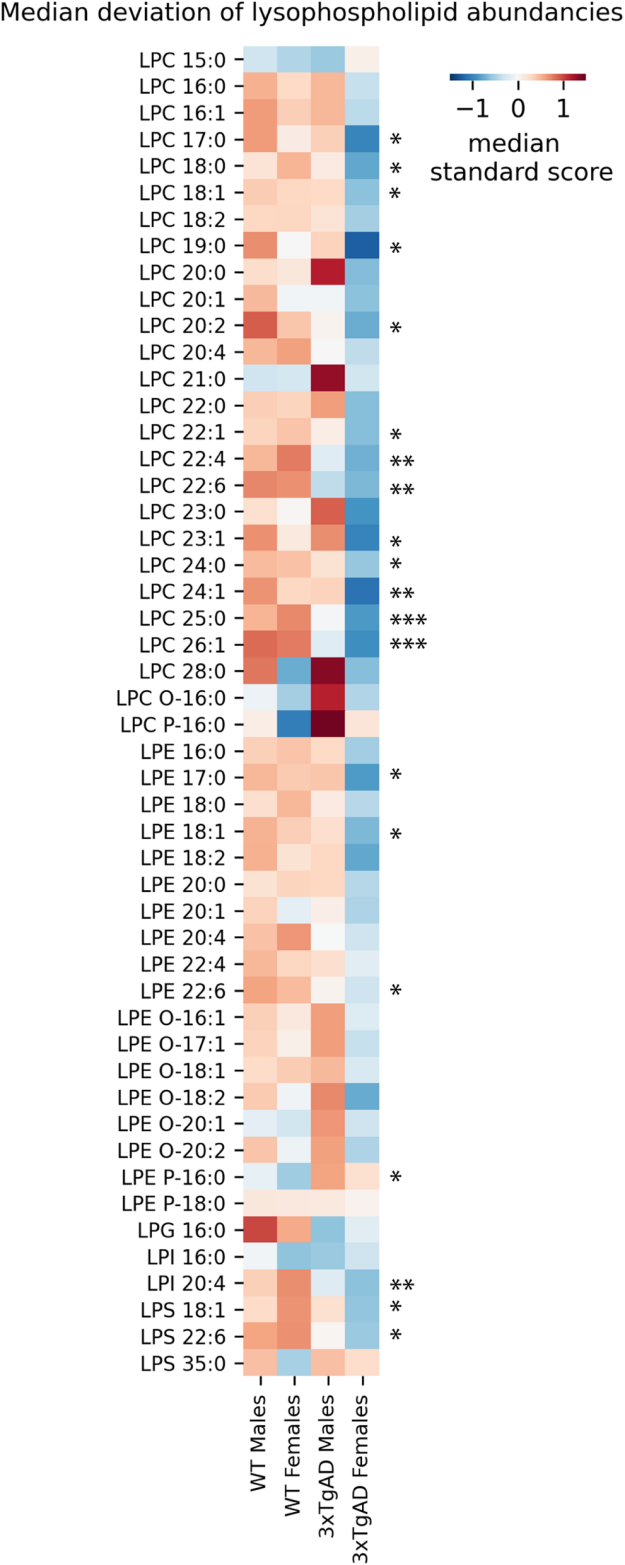
Sex-specific genotype-related differences in lysophospholipids. Heatmap of median standard scores of lysophospholipid species abundance in each sample group. Asterisks next to the plot denote significant *p*-values for a genotype effect, less than 0.05 (*), less than 0.01 (**), or less than 0.001 (***). LPC, lysophosphatidylcholine; O, ether linkage; P plasmalogen linkage; LPE, lysophosphatidylethanolamine; LPG, lysophosphatidylglycerol; LPI, lysophosphatidylinositol; LPS lysophosphatidylserine; M, male; F, female; WT, wild-type; 3xTg-AD, triple transgenic Alzheimer’s disease model

To elucidate alterations (and potentially sex biases) in lipid metabolism, we performed lipid ontology analysis on molecular species exhibiting a genotype effect. According to enrichment analysis, long-chain free fatty acids (FFAs) and lysophospholipids (LPLs) were significantly affected in association with the AD phenotype (Figs. 5 and 6, Table S5). In addition, analysis of proteins involved in lipid metabolic pathways also supported the observed changes at the metabolite level (Fig. 5, Figure S4). While not all individual FFA species reached significance, both total long-chain free fatty acids and the enzymes involved in early fatty acid elongation were depleted in transgenic animals (Fig. 5) (genotype effect p_Total FFA_ = 0.009, p_Hadhb_ = 0.005, p_Hadh_ = 0.068 (ns), p_Hadha_ = 0.024, p_Echs1_ = 2.0E^−5^, p_Mecr_ = 0.001, p_Ppt1_ = 4.2E^−11^). One long-chain fatty acid transporter was also significantly decreased in transgenic animals (genotype effect p_Slc27a1_ = 2.2E^−7^) (Figure S4).

Lysophospholipids were also enriched based on a genotype effect, however, closer observation revealed that a decrease in transgenic animals was driven by females only (Fig. 6). LPLs and in particular lysophatidylcholines followed a consistent trend of decreased levels in female 3xTg-AD only (Fig. 6). Total lysophospholipids in female 3xTg-AD were significantly lower compared to the other groups (p_Total LPL_ = 0.019). We further explored the proteins involved in LPL metabolism and found two specific enzymes, Lclat1 and Enpp2, that also exhibited a significant genotype effect driven by females (Figure S4) (genotype effect p_Lclat1_ = 0.021, p_Enpp2_ = 0.049).

## Discussion

To characterize the metabolic changes underlying AD and sex biases therein, we applied an untargeted discovery approach for broad coverage of the polar metabolome, lipidome, and proteome without a priori hypotheses. By using multiple layers of data that complement each other, data-driven hypotheses generated from one omics type can be cross-validated by another and we can obtain additional determinant insights into altered metabolism [37, 38]. To facilitate data integration and limit variability between these datasets, we developed a sequential extraction protocol to recover all omics data layers from the same sample, as opposed to using independent samples (Fig. 1). While biphasic methods exist to simultaneously extract both polar and lipid metabolites, single-phase methods tailored to each are more reproducible and effective [65–69]. This is particularly true for amphipathic lipids which can be lost through biphasic extractions as they diffuse and partition between both phases. Therefore, we performed two consecutive single-phase extractions with methanol:water (4:1, v/v) and 1-butanol:methanol (1:1, v/v) and pooled both extracts for lipid analysis (Fig. 1). Since proteins are essentially insoluble in pure organic solvents, the precipitated protein pellet can be lysed and reconstituted for proteomics analysis, allowing for extraction of a wide range of proteins from the same sample (Fig. 1). A combined acquisition using high-resolution MS^1^ and MS^2^ (MS/MS) data allowed for metabolite annotation with a high level of confidence. While the quality and quantity of the acquired MS/MS data is limited by the instrument sensitivity and scanning speed (in addition to available spectral libraries), the acquisition of multiple data layers compensates to a certain extent for this type of inherent bias, since protein data allow to bridge the gaps in the segments of the pathways where metabolite information is lacking, or vice versa [70]. Using an integrative multivariate analysis approach (i.e., MOFA), the acquired comprehensive metabolite and protein signatures of brain tissue revealed both AD-associated alterations and disease-related sex differences in brain metabolism. The identification of sex differences at the molecular level is essential to understand the molecular mechanisms behind sex biases observed in AD prevalence and symptoms [9, 71–76]. To gain understanding on the biochemical and physiological relevance of revealed metabolic alterations, the data were further explored in the context of metabolic pathways and lipid ontology, thereby highlighting AD-related sex biases in amino acid and lysophospholipid metabolism and deregulated free fatty acid elongation in the AD brain (Figs. 3, 4, and 5).

### Alterations in amino acid metabolism in AD are sex-dependent

Within alanine, aspartate, and glutamate metabolism, we found two coordinated hubs of sex-biased alterations in AD (Fig. 3). The alanine, aspartate, and glutamate pathway is connected to various cellular processes including energy metabolism and the biosynthesis of neurotransmitters. Sex biases within this pathway can therefore influence multiple aspects of cell behavior and health, and by extension AD pathology. Multiple alterations in alanine, aspartate, and glutamate metabolism in AD have been previously reported [29, 32, 77–79] and correlated to key AD pathologies [29]. As discussed in further detail below, some of these changes can be directly linked to cognitive function.

At the entrance to the alanine, aspartate, and glutamate pathway lies NAAG, which is transformed into N-acetyl-aspartate (NAA) and finally aspartate (Fig. 3). NAAG is a neurotransmitter with a role in cognition while its product NAA is a marker of viable neurons [80, 81]. Previously reported reduction in both NAAG and NAA in AD can indicate both impaired cognition and neuronal loss [81–83]. Our findings showed in addition that NAAG and NAA levels were particularly depleted in 3xTg-AD males, suggesting worsened cognitive function compared to the females (Fig. 3). These results at the metabolite level were further supported by a significant downregulation of the Folh1 enzyme, which catalyzes the transformation of NAAG into NAA, in 3xTg-AD males (compared to WT) (Fig. 3), although this enzyme was differentially expressed in males and females regardless of genotype. These findings might imply a compensatory mechanism to reduce the catabolism of NAAG and preserve the necessary amounts for brain function. The potential of different mechanisms underlying loss of cognitive function in AD between each sex, such as sex-hormone-driven effects, must be further explored as they might have important implications for prognosis and presumed therapeutic strategies.

Another neurotransmitter produced through glutamate metabolism is GABA. GABA has been previously reported to be increased in the AD brain and linked to impaired cognition and increased reactive astrocytes surrounding amyloid plaques, as reviewed elsewhere [84]. While we did not find any significant changes in total GABA levels, multiple enzymes and intermediates in different GABA synthesis routes were altered (Fig. 3). This observation of unchanging GABA levels might be due to our analysis of the whole brain, where some areas or cell types may compensate for each other. Indeed, in healthy brain tissue GABA will be produced by GABAergic neurons; however, if these die reactive astrocytes can compensate by producing GABA from putrescine via MAO-B [85, 86]. Our results provide partial evidence of this manner of compensation by reactive astrocytes, since we found a significant increase in the intermediate metabolite of this pathway, N-acetyl-GABA, in 3xTg-AD animals. This would suggest a hyperactivation of this pathway in AD and could also explain the lack of change in total GABA levels. However, we did not find any significant changes in the expression levels of MAO-B (Table S1), although this does not necessarily indicate a lack of change in its activity.

We have in addition found a sex difference within GABA synthesis in AD; enzymes Abat and Gad1 were significantly depleted in 3xTg-AD females. It would make sense for the compensatory pathway to be further upregulated in this case, and we do see a more dramatic increase in N-acetyl-GABA in this population, although it did not reach significance for an interaction effect. One study has provided evidence for a stronger increase of GABA in murine AD females compared to males and linked it to progressive cognitive decline [87]. Clearly, the potential of sex differences in GABA synthesis (perhaps involving reactive astrocytes) could benefit from further research.

Reactive astrocytes may also be implicated based on our findings related to arginine metabolism. We have observed significantly increased arginine levels in 3xTg-AD females (Fig. 4). Accumulation of arginine in AD has previously been reported in the mouse brain and in both the human brain and plasma [27, 30, 32, 88, 89]. Furthermore, arginine accumulation has been linked to inflammation, another hallmark of AD [1, 90]. Previous studies have reported the accumulation, and later release, of arginine by astrocytes in inflammatory environments to reduce the production of free radicals by Nos [88, 90]. However, to our knowledge, our study is the first report of sex-biased arginine increase in AD. We can therefore speculate on the possibility of sex-biased phenotypes within reactive astrocytes which have recently been placed in the center of AD pathology as an early event (i.e., reactive astrogliosis) in AD progression [91, 92]. This would not be so surprising, since sex differences in glial cells have been previously described, reviewed elsewhere [93], and were recently also reported in microglial cells in AD [94].

Arginine biosynthesis in the brain is also linked to ammonia waste management, as the vast majority of ammonia is incorporated into glutamine by Glul and transported in this way to the liver for disposal via the urea cycle [90, 95]. We found that Glul expression was significantly decreased in the 3xTg-AD brain compared to WT (Fig. 4). This result is in accordance with previous reports on the failure of ammonia elimination mechanisms in AD [95, 96]. In normal conditions, the urea cycle in the brain is not complete due to the lack of Otc enzyme expression, so Glul is essential for the removal of ammonia. However, in AD, ammonia was shown to accumulate [95, 96]. The excess of ammonia has been linked to impaired cognition and memory, increased blood-brain barrier permeability, and diminished energy metabolism [95]. Unable to deal with the excessive ammonia, Otc expression can be induced in cerebral vascular endothelial cells, creating a complete urea cycle [97]. However, our results did not reveal the increase in Otc expression in 3xTg-AD mouse brain.

### Alterations in lipids imply the link to inflammation in the AD brain

Our study found an overall decrease in long-chain FFAs (regardless of saturation state) in the 8-month 3xTg-AD brain compared to WT (Fig. 5A). In addition, protein enrichment analysis showed that the levels of proteins involved in fatty acid elongation and one long-chain FFA transporter (Scl27a1) from the Slc27a family were significantly downregulated in the 3xTg-AD brain (Fig. 5B, Figure S4). These findings imply that FFA transport into and synthesis within AD brains might be altered. Previous reports of FFA levels in AD in both the blood and in the brain have been contradictory, as both accumulation and depletion were observed in humans and mice [33, 79, 98–104]. One plausible explanation for these reported differences might be variable alterations in FFA levels with the stage of disease progression. Given the strong associations previously revealed between FFA levels and memory, neuronal signaling (e.g., long-term potentiation, neurotransmitter release), and inflammation, FFA metabolism might have an essential role in AD pathology [105–108].

Lysophospholipids, and notably their most abundant subclasses lysophosphatidylcholines and lysophosphatidylethanolamines, have been widely reported to be decreased in AD [26, 102, 109–112]. Our study further uncovered a sex bias; we found that the observed decrease was driven by females (Fig. 6). LPLs have been recognized as important bioactive molecules that participate in multiple signaling cascades and can remodel cellular membranes [113, 114]. Moreover, decreased lysophosphatidylcholine has previously been correlated to amyloid β load [112]. To explore the mechanisms underlying LPL decrease in 3xTg-AD females, we further focused on the enzymes involved in LPL metabolism. A primary pathway of LPL metabolism is through the Land’s cycle. Phospholipases A cleave glycerophospholipids into a FFA and an LPL, while lysophospholipid acyltransferases (LPAT) catalyze the opposite reaction. Previous studies have suggested decreased LPLs to be caused by decreased phospholipase A2 activity and increased LPAT activity [115, 116]. We did not find phospholipase levels to be decreased in female 3xTg-AD mice. However, one LPAT enzyme, Lclat1, had significantly higher expression in 3xTg-AD females and therefore could explain the LPL sex bias that we observed (Figure S4). Alternatively, beyond the Land’s cycle, LPLs can be degraded by phospholipases. We found a significant increase in the expression of phospholipase Enpp2 in 3xTg-AD mice which was, once again, driven by females (Figure S4). If Enpp2 is indeed more active, more of the lysophosphatidic acid will be produced. This hypothesis has interesting potential because lysophosphatidic acid has been associated with Alzheimer’s pathology biomarkers [117], the disruption of neurotransmission, and increased permeability of the blood-brain barrier [109, 118–120]. Further efforts will be needed to untangle lysophospholipid metabolism in the AD brain and its implications.

### Limitations

Our study is exploratory in nature, relying on global untargeted approaches and acquired omics data with the aim to generate data-driven hypotheses. These hypotheses need to be further investigated using orthogonal and targeted experimental approaches with statistical rigor. In addition, the untargeted analyses were performed on a homogenate of a whole brain tissue which presents some limitations. Different brain regions and cell types can have different metabolic profiles and activity, therefore our results reflect an averaged effect across many different cell types and structurally and functionally distinct regions. Finally, at 8 months this mouse model does not yet exhibit amyloidẞ plaques and neurofibrillary tangles, the well-established pathological markers of AD [44]. The metabolic profile may further change as the disease advances and tangles appear.

## Conclusion

Through multi-omics data integration and pathway-level analyses, we have identified several areas of altered metabolism in AD. Moreover, we have highlighted metabolic sex biases within AD that may contribute to explaining the known differences in disease prevalence and symptoms. This exploratory data-driven study provides a starting point for further research into mechanisms of impaired cognition that may be related to different neurotransmitters for each sex, into the role of ammonia waste management in the AD brain, the relationship between AD and oxidative stress initiated by NOS, the roles of long-chain FFA in memory, and the relevance of LPLs and lysophosphatidic acid metabolism in the development of AD in females.

## Supplementary Information

**Additional file 1: **Supplementary information about materials and methods – detailed description of untargeted analysis of polar metabolites, lipids and proteins. Supplementary figures: **Figure S1.** Analysis of collision energies to result in maximum identification of polar and lipid metabolites. **Figure S2.** Effects of omics data and contributions to MOFA factors. **Figure S3.** Additional measured metabolites (beige background) and proteins (grey background, bold title) from the alanine, aspartate, and glutamate metabolic pathway. **Figure S4.** Additional proteins involved in lipid metabolism. (A) Free fatty acid transporter Slc27a1. (B) Enzymes Lclat1 and Epp1 involved in lysophospholipid metabolism. **Figure S5*.*** Supplementary proteomics data. (A) Boxplots of raw protein quantities before 3-precursor filtering. (B) Principal component analysis on proteomics data (see also Supplementary Table S4).

**Additional file 2: **List of all compounds identified through proteomics (**Table S1**), metabolomics (**Table S2**), or lipidomics (**Table S3**) analyses by MS/MS spectral matching, including results of ANOVA analysis on each identified compound for genotype, sex, or sex:genotype interaction effects. **Table S4.** Results output of MetaboAnalyst joint pathway analysis. **Table S5.** Results output of LION lipid ontology analysis using lipids ranked according to genotype effect, calculated using either a RankSum or a Fisher Exact Test analysis. **Table S6.** Supplementary table of metabolomics data from untargeted mass spectrometry analysis, normalized to [mg] tissue weight. **Table S7.** Supplementary table of lipidomics data from untargeted mass spectrometry analysis, normalized to [mg] tissue weight. **Table S8.** Supplementary table of proteomics data from untargeted mass spectrometry analysis.

## Data Availability

The mass spectrometry proteomics data have been deposited to the ProteomeXchange Consortium via the PRIDE [121] partner repository with the dataset identifier PXD033164. The mass spectrometry polar metabolomics and lipidomics data have been deposited into the MetaboLights repository with the dataset identifier MTBLS4812 [122].

## Abbreviations

3xTg-AD: Triple-transgenic Alzheimer’s disease mouse model
AD: Alzheimer’s disease
ANOVA: Analysis of variance
BuMe: 1-butanol:methanol
ESI: Electrospray ionization
FFA: Free fatty acid
GABA: Gamma-aminobutyric acid
HILIC: Hyrophilic-interaction chromatography
HRMS: High-resolution mass spectrometry
LOWESS: Locally weighted scatterplot smoothing
LPAT: Lysophospholipid acyltransferases
LPL: Lysophospholipid
MOFA: Multi-omics factor analysis
NAA: N-acetyl-aspartate
NAAG: N-acetyl-aspartyl-glutamate
QC: Quality control
RPLC: Reversed-phase chromatography
WT: Wild-type
