# Supplementary material for: Molecular insights into sex-specific metabolic alterations in Alzheimer’s mouse brain using multi-omics approach
Source: Alzheimers Res Ther. 2023 Jan 9;15:8. doi: 10.1186/s13195-023-01162-4 (PMC9827669; doi:10.1186/s13195-023-01162-4)
Supplement: Supplementary file 1 — Additional file 1: Supplementary information about materials and methods – detailed description of untargeted analysis of polar metabolites, lipids and proteins. Supplementary figures: Figure S1. Analysis of collision energies to result in maximum identification of polar and lipid metabolites. Figure S2. Effects of omics data and contributions to MOFA factors. Figure S3. Additional measured metabolites (beige background) and proteins (grey background, bold title) from the alanine, aspartate, and glutamate metabolic pathway. Figure S4. Additional proteins involved in lipid metabolism. (A) Free fatty acid transporter Slc27a1. (B) Enzymes Lclat1 and Epp1 involved in lysophospholipid metabolism. Figure S5. Supplementary proteomics data. (A) Boxplots of raw protein quantities before 3-precursor filtering. (B) Principal component analysis on proteomics data (see also Supplementary Table S4). [file 13195_2023_1162_MOESM1_ESM.docx]

# **Additional file 1:** Supplementary Information for Publication

# Supplementary information about materials and methods

## Untargeted LC-HRMS Metabolomics

Global-untargeted metabolite profiling was performed by HILIC chromatography coupled to high-resolution mass spectrometry (HRMS) operating in both positive and negative (ESI+ and ESI-) mode on 6550 iFunnel Q-TOF mass spectrometer interfaced with 1290 UHPLC system (Agilent Technologies). Samples were analyzed using two chromatographic separations: i) BEH Amide, 1.7 μm, 100 mm × 2.1 mm I.D. column (Waters, Massachusetts, US) in positive ionization mode and, ii) SeQuant® ZIC-pHILIC, 5 μm, 100 mm × 2.1 mm I.D. column (Merck, Darmstadt, Germany) with a SeQuant® ZIC-pHILIC, 5 μm, 20 mm × 2.1 mm I.D. guard column (Merck, Darmstadt, Germany) in negative ionization mode. The column temperature was maintained constant at 25°C and 30°C in positive and negative ionization mode, respectively. The mobile phase was composed of A = 20mM ammonium formate and 0.1% formic acid in water (pH 3.8) and B = 0.1% formic acid in 100% ACN for positive mode and A = 20mM ammonium acetate and 20mM ammonium hydroxide in water (pH 9.3) and B = 100% ACN for negative mode. In positive mode, the linear elution gradient from 95% B (0-1.5 min) to 45% B (17-19 min) was applied. The initial gradient conditions were restored within one minute and a 5-min post run re-equilibration was applied to maintain the system reproducibility. In negative mode, the linear stepwise elution gradient from 90% B (0-1.5 min) to 50% B (8-11 min) to 45% B (12-15 min) was applied. The initial gradient conditions were restored within one minute and a 9-min post-run equilibration was applied to maintain the system reproducibility. The flow rates were 400 μL/min and 300 μL/min in positive and negative ionization mode, respectively. In both cases, the sample injection volume was 2 µl. ESI source conditions were set as follows: dry gas temperature 290 °C and flow 14 L/min, fragmentor voltage 380 V, sheath gas temperature 350 °C and flow 12 L/min, nozzle voltage 0 V, and capillary voltage +2000 V in positive mode and -2000 V in negative. The instrument was set to acquire over the full m/z range 50-1200 in both modes, with the MS acquisition rate of 2 spectra/s. Iterative MS/MS data dependent scan acquisition mode was applied for polar metabolite identification and annotation. Five iterations were performed with rolling exclusion over the m/z range of 30-1200 at a rate of 3 spectra/s and a narrow isolation width (~1.3 amu). A combined collision energy of 10 and 25 eV was used in positive ionization mode, and 10 eV in negative mode.

## Untargeted LC-HRMS Lipidomics

Extracted samples were analysed by reversed phase liquid chromatography coupled to a high-resolution mass spectrometry (RPLC-HRMS) instrument (Agilent 6550 IonFunnel QTOF). In both, positive and negative ionisation mode, the chromatographic separation was carried out on a Zorbax Eclipse Plus C18 (1.8 μm, 100 mm × 2.1 mm I.D. column) (Agilent technologies, USA). Mobile phase was composed of A = 60:40 (v/v) Acetonitrile:water with 10 mM ammonium acetate and 0.1% acetic acid and B = 88:10:2 Isopropanol:acetonitrile:water with 10 mM ammonium acetate and 0.1% acetic acid. The linear gradient elution from 15% to 30% B was applied for 2 minutes, then from 30% to 48% B for 0.5 minutes, from 48% to 72% B and last gradient step from 72% to 99% B followed by 0.5 minutes isocratic conditions and a 3 min re-equilibration to the initial chromatographic conditions. The flow rate was 600 μL/min, column temperature 60 °C and sample injection volume 2 μL.

ESI source conditions were set as follows: dry gas temperature 200 °C, nebulizer 35 psi and flow 14 L/min, sheath gas temperature 300 °C and flow 11 L/min, nozzle voltage 1000 V, and capillary voltage +/- 3500 V. Full scan acquisition mode in the mass range of 100-1700 *m/z* was applied for data acquisition while iterative MS/MS data dependent scan acquisition mode was applied for lipid identification and annotation. Five iterations were performed with rolling exclusion over the m/z range of 30-1200 at a rate of 3 spectra/s and a narrow isolation width (~1.3 amu). A combined collision energy of 25 and 40 eV was used in both positive and negative ionization modes.

## Untargeted LC-HRMS Proteomics

### Supplementary Data

In total, 80668 precursors in the library were quantitated with the DIA data (cumulative on all runs). These corresponded to 66528 peptides, resulting in **6742** inferred protein groups. Precursor mass tolerances used were 10.5 and 10.9 ppm in MS1 and MS2, respectively. Average mass errors were 3.02 and 2.99 ppm in MS1 and MS2, respectively. The average of data points per LC peak was 8 in both MS1 and MS2.

The dataset was filtered further to retain only protein groups that were quantitated in all samples with minimum average of 3 precursors. (**5115 proteins**, see Supplementary Table S4 and Supplementary Figure S5).

### Tissue extraction

Homogenized tissue material after solvent extraction was allowed to dry under air flow 5 min in the homogenization tubes under a laminar flow hood. The pellet was resuspended in lysis buffer (1% Sodium deoxycholate, 30 mM Tris pH 8.6, 10 mM DTT) with a ratio of 150 µL/ 10 mg of initial tissue weight and shaken in the FastPrep system for 3x20s, with 3 min cooling on ice in between runs. An aliquot of 300 µL was taken, mixed 1:1 (v/v) with lysis buffer, heated at 95C for 10 min with shaking and used for all subsequent steps.

### Protein Digestion

Aliquots of samples were digested following a modified version of the iST method (Kulak et al., 2014). Briefly, 100 µg of proteins at 2 µg/µL, based on tryptophane fluorescence quantification (Wisniewski et al., 2015) were transferred to new tubes. Samples were then diluted 1:1 (v:v) with water containing 4 mM MgCl_2_ and Benzonase (Merck #70746, 100x dil of stock = 250 Units/µL) and incubated for 15 minutes at RT to digest nucleic acids. Reduced disulfides were alkylated by adding ¼ vol of 160 mM chloroacetamide (final 32 mM) and incubating at 25°C for 45 min in the dark. Samples were adjusted to 3 mM EDTA and digested with 1 µg Trypsin/LysC mix (Promega #V5073) under gentle shaking for 1h at 37°C, followed by a second 1h digestion with a second, identical aliquot of proteases. To remove sodium deoxycholate, two sample volumes of isopropanol containing 1% TFA were added to the digests, and the samples were desalted on a cation exchange plate (Oasis MCX microelution plate; Waters Corp., Milford, MA, prod.#186001830BA) by centrifugation. After washing with isopropanol/1%TFA, peptides were eluted in 250 µL of 80% MeCN, 19% water, 1% (v/v) ammonia. Eluates after SCX desalting were dried, and resuspended in 100 µL of 2% MeCN, 0.1% TFA.

### Peptide fractionation for library construction

Aliquots of 5 µg of all samples were mixed to create a pool, which was manually separated into 7 fractions by off-line basic reversed-phase (bRP) using the Pierce High pH Reversed-Phase Peptide Fractionation Kit (Thermo Fisher Scientific). The fractions collected were: flow through, 7.5, 10, 12.5, 15, 17.5 and 50% MeCN in 0.1 % triethylamine (~pH 10). Dried bRP fractions were redissolved in 100 µL buffer A and 5 µL were injected for LC-MS/MS analysis.

### Liquid Chromatography-Mass spectrometry

LC-MS/MS analysis was carried out on a TIMS-TOF Pro (Bruker, Bremen, Germany) mass spectrometer interfaced through a nanospray ion source (“captive spray”) to an Ultimate 3000 RSLCnano HPLC system (Dionex). Peptides were separated on a reversed-phase custom packed 40 cm C18 column (75 μm ID, 100Å, Reprosil Pur 1.9 µm particles, Dr. Maisch, Germany) at a flow rate of 0.250 µL/min with a 6-27% acetonitrile gradient in 92 min followed by a ramp to 45% in 15 min and to 95% in 5 min (all solvents contained 0.1% formic acid). Identical LC gradients were used for DDA and DIA measurements.

For creation of the spectral library, data-dependent acquisition (DDA) was carried out on the 7 bRP fractions using a standard TIMS PASEF method (Meier et al 2018) with ion accumulation for 100 ms for each the survey MS1 scan and the TIMS-coupled MS2 scans. Duty cycle was kept at 100%. Up to 10 precursors were targeted per TIMS scan. Precursor isolation was done with *m/z* windows of 2 or 3 units, below or above m/z 800, respectively. The minimum threshold intensity for precursor selection was 2500. If the inclusion list allowed it, precursors were targeted more than one time to reach a minimum target total intensity of 20000. Collision energy was ramped linearly based uniquely on the 1/k_0_ values from 20 (at 1/k_0_=0.6) to 59 eV (at 1/k_0_=1.6). Total duration of a scan cycle including one survey and 10 MS2 TIMS scans was 1.16 s. Precursors could be targeted again in subsequent cycles if their signal increased by a factor 4.0 or more. After selection in one cycle, precursors were excluded from further selection for 60 s. Mass resolution in all MS measurements was approximately 35000.

The diaPASEF method used mostly the same instrument parameters as the DDA methods and was as reported previously (Meier et al 2020). Per cycle, the mass range 400-1200 m/z was covered by a total of 32 windows, each 26 *m/z* units wide (overlap of 1 m/z) and a 1/k_0_ range of 0.3. Collision energy and resolution settings were the same as in the DDA method. Two windows were acquired per TIMS scan (100 ms) so that the total cycle time was 1.7 s.

### Library creation

Raw Bruker MS data were processed directly with Spectronaut 14.10 (Biognosys, Schlieren, Switzerland). A library was constructed from the DDA data for the fractions by searching the reference mouse proteome (RefProt, [www.UNIPROT.org](http://www.UNIPROT.org)) database of August 26^th^, 2020 (55485 sequences). For identification, peptides of 7-52 AA length were considered, cleaved with Trypsin/P specificity and a maximum of 2 missed cleavages. Carbamidomethyl-Cys (fixed), Met oxidation and N-terminal protein acetylation (variable) were the modifications applied. Mass calibration was dynamic and based on a first database search. The Pulsar engine was used for peptide identification. Protein inference was performed with the IDPicker algorithm. PSM, peptide and protein identifications were all filtered at 1% FDR against a decoy database.

Specific filtering for library construction filtered out fragments corresponding to less than 3 AA and fragments outside the 300-1800 m/z range. Also, only fragments with a minimum base peak intensity of 5% were kept. Precursors with less than 3 fragments were also eliminated and only the best 6 fragments were kept per precursor. For quantitation only PSMs with maximum one missed cleavage were used. No filtering was done based on charge state. Shared (non proteotypic) peptides were kept.

The library created contained 106942 precursors mapping to 81650 stripped sequences, of which 33638 were proteotypic. These corresponded to 7951 protein groups (11475 proteins). Of these, 11934 were single hits (one PSM). In total 624880 fragments were used for quantitation.

### DIA quantitation

Peptide-Centric analysis of DIA data was done with Spectronaut 14.10 using the library described above. Both MS1 and MS2 data were used for quantitation (Huang et al 2020). Run alignment was assisted by a deep learning algorithm based on sample-specific in silico prediction of retention times of identified peptides and alignment based on local nonlinear regression. Interference correction (from neighboring isotope envelopes) was performed at both the MS1 and MS2 levels using windows of 2 and 3 min respectively. Single hits proteins (defined as matched by one stripped sequence only) were kept in the Spectronaut analysis. Peptide quantitation was based on XIC area, for which a minimum of 1 and a maximum of 3 (the 3 best) precursors were considered for each peptide, from which the median value was selected. Peptides were retained for calculation of protein group quantities if they passed the set Q-value threshold of identification (0.01) in at least 50% of the runs. For the retained peptides, any missing values were imputed with low-shifted values based on the global distribution of values in the entire experiment. Quantities for protein groups were obtained by summing all assigned peptide intensities after filtering. Global normalization of runs/samples was done based on the median of peptides.

### Data processing and statistical tests

All subsequent analysis was done with the Perseus software package (Tyanova et al 2016). Intensity values were log_2_-transformed. For subsequent steps only protein groups quantitated with an average (across all samples) of 3 precursors were kept (5116 proteins). After assignment to groups, a Welch t-tests were carried out among all conditions, with Benjamini-Hochberg correction for multiple testing (Q-value threshold >0.05). The difference of means obtained from the test were used for 1D enrichment analysis on associated GO/KEGG annotation as described (Cox and Mann, 2012). The enrichment analysis was also FDR-filtered (Benjamini-Hochberg , Q-val<0.02).

All raw MS data together with raw output tables are available via the Proteomexchange data repository ([www.proteomexchange.org](http://www.proteomexchange.org)) with the accession PXD033164.

---------------------------

## References

Kulak, N. A., Pichler, G., Paron, I., Nagaraj, N., Mann, M. (2014). Minimal, encapsulated proteomic-sample processing applied to copy-number estimation in eukaryotic cells. Nature Methods 11(3): 319–24.

Wisniewski, J.R., Gaugaz, F.Z. (2015) Fast and Sensitive Total Protein and Peptide Assays for Proteomic Analysis. Anal. Chem. 87(8): 4110-4116.

Meier, F., Brunner, A., Koch, S., Koch, H., Lubeck, M., Krause, M., … Mann, M. (2018). Online Parallel Accumulation-Serial Fragmentation (PASEF) with a Novel Trapped Ion Mobility Mass Spectrometer. Molecular & Cellular Proteomics : MCP, 17(12), 2534–2545. https://doi.org/10.1074/mcp.TIR118.000900

Meier, F., Brunner, A.-D., Frank, M., Ha, A., Bludau, I., Voytik, E., … Mann, M. (2020). diaPASEF: parallel accumulation-serial fragmentation combined with data-independent acquisition. Nature Methods, 17(12), 1229–1236. https://doi.org/10.1038/s41592-020-00998-0

Bruderer, R., Bernhardt, O. M., Gandhi, T., Miladinović, S. M., Cheng, L.-Y., Messner, S., … Reiter, L. (2015). Extending the limits of quantitative proteome profiling with data-independent acquisition and application to acetaminophen-treated three-dimensional liver microtissues. Molecular & Cellular Proteomics : MCP, 14(5), 1400–1410. <https://doi.org/10.1074/mcp.M114.044305>

Huang, T., Bruderer, R., Muntel, J., Xuan, Y., Vitek, O., & Reiter, L. (2020). Combining precursor and fragment information for improved detection of differential abundance in data independent acquisition*□s. Molecular and Cellular Proteomics, 19(2), 421–430. <https://doi.org/10.1074/mcp.RA119.001705>

Benjamini, Y., Hochberg, Y. (1995) Controlling the false discovery rate: a practical and powerful approach to multiple testing. J. R. Stat. Soc. Ser. B 57: 289–300.

Tyanova, S., Temu, T., Sinitcyn, P., et al. (2016) The Perseus computational platform for comprehensive analysis of (prote)omics data. Nat Methods 13(9):731–740.

Cox, J., & Mann, M. (2012). 1D and 2D annotation enrichment: a statistical method integrating quantitative proteomics with complementary high-throughput data. BMC Bioinformatics, 13 Suppl 1(Suppl 16), S12. <https://doi.org/10.1186/1471-2105-13-S16-S12>

# Supplementary figures


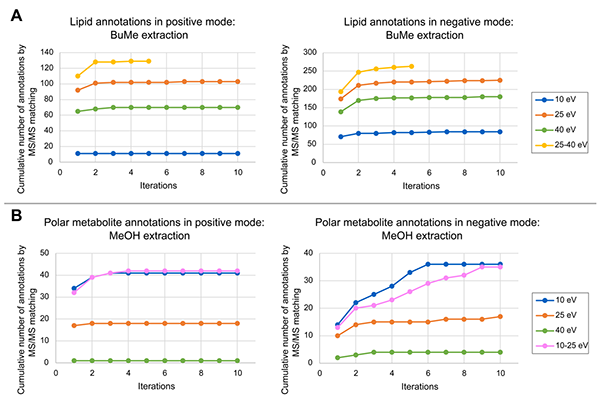


**Figure S1.** Analysis of collision energies to result in maximum identification of polar and lipid metabolites. MS/MS data were acquired in multiple collision energy conditions over up to ten iterations of data-dependent acquisition with rolling exclusion. (A) The cumulative number of lipids identified based on MS/MS spectral matching to the LipidBlast database. Lipids were extracted from sample homogenate using 1-butanol:methanol (BuMe, 1:1 v/v). (B) The cumulative number of polar metabolites identified based on MS/MS spectral matching to the Fiehn-HILIC database. Polar metabolites were extracted from sample homogenate using 80% methanol. *MS/MS: tandem mass spectrometry*.


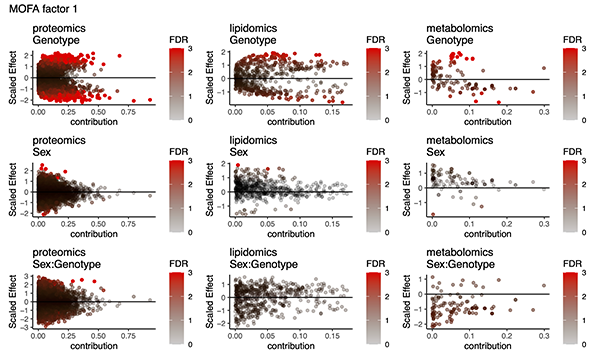


**Figure S2**. Effects of omics data and contributions to MOFA factors. Each spot is a single compound placed according to it's contribution to a given MOFA factor and it's scaled genotype, sex, or sex:genotype interaction effect. Spots are colored according to the FDR-corrected p-value. Results are shown for all compounds identified, respectively, by proteomics, lipidomics, and metabolomics. *FDR: false discovery rate*.


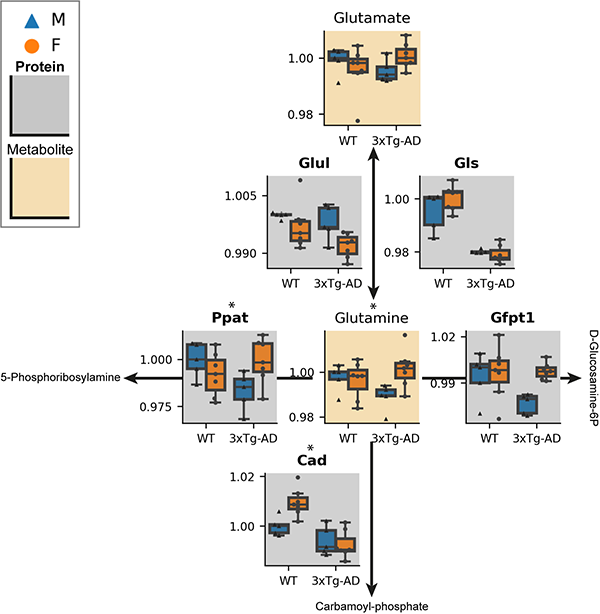


**Figure S3.** Additional measured metabolites (beige background) and proteins (grey background, bold title) from the alanine, aspartate, and glutamate metabolic pathway are represented by boxplots. Each line of the boxplot is a quartile of the compound abundancy per sample group (relative to the median value of male WT). Boxes are colored blue or orange with triangular or circular points for males and females, respectively. Asterisks above plots denotate significant p-values for a sex:genotype interaction effect, less than 0.05 (*) or less than 0.01 (**). *M: male; F: female; WT: wild-type; 3xTg-AD: triple transgenic Alzheimer's disease model.*


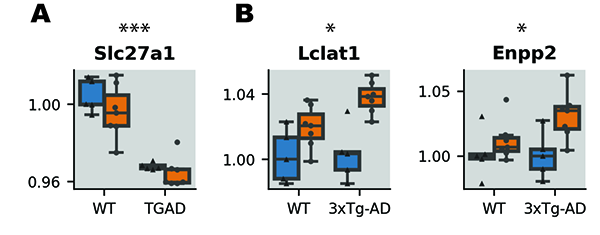


**Figure S4**. Additional proteins involved in lipid metabolism. Proteins are represented by boxplots where each line of the boxplot is a quartile of the compound abundancy per sample group (relative to the median value of male WT). Boxes are colored blue or orange with triangular or circular points for males and females, respectively. (A) Free fatty acid transporter Slc27a1. (B) Enzymes Lclat1 and Epp1 involved in lysophospholipid metabolism. Asterisks above plots denotate significant p-values for genotype effect, less than 0.05 (*) or less than 0.001 (***). *M: male; F: female; WT: wild-type; 3xTg-AD: triple transgenic Alzheimer's disease model.*


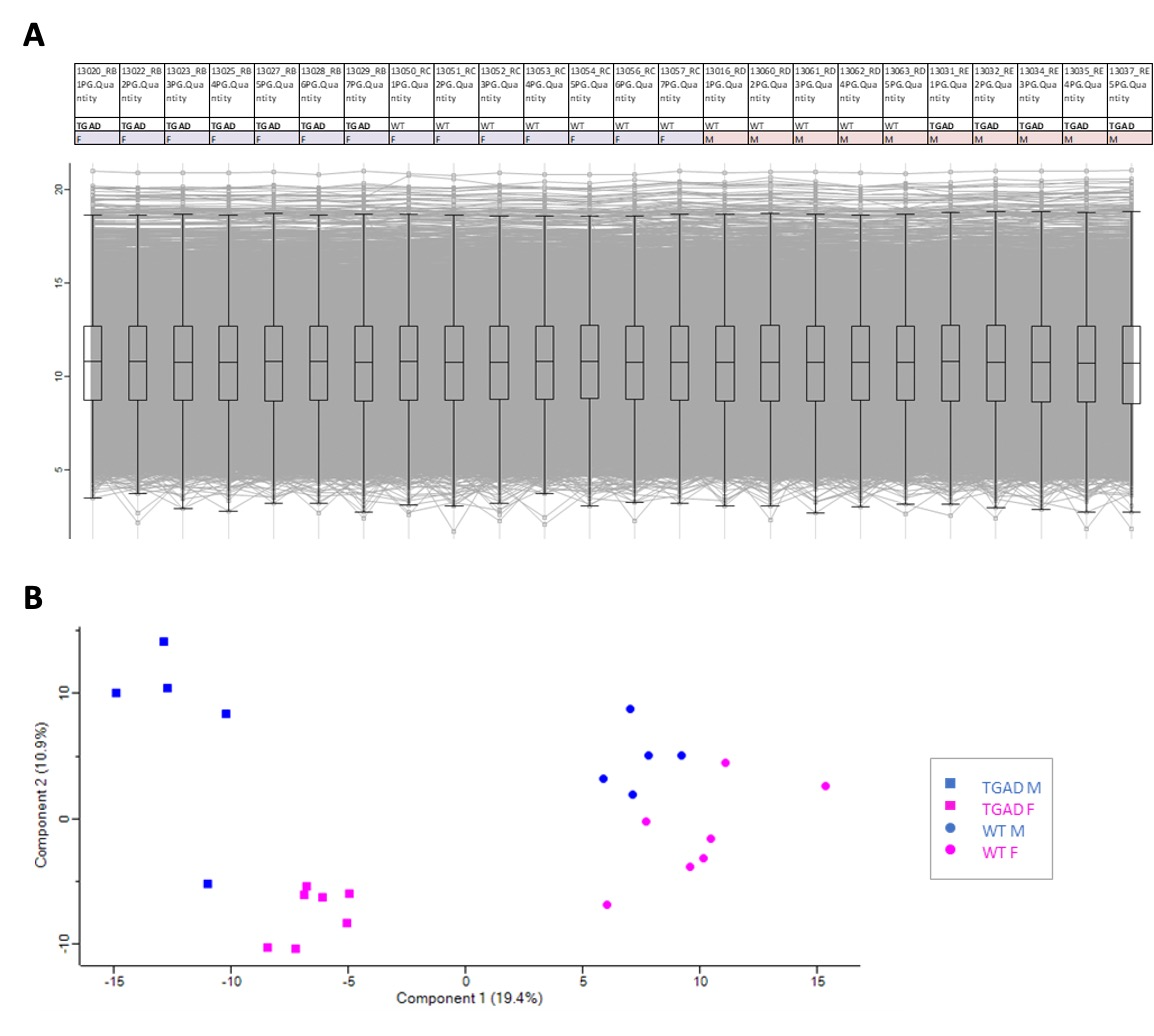


**Figure S5*.*** Supplementary proteomics data. (A) Boxplots of raw protein quantities before 3-precursor filtering. (B) Principal component analysis on proteomics data (see also Supplementary Table S4). *TGAD: triple-transgenic Alzheimer's disease model; M: male; F:female; WT wild-type*.
